# Annotations

**Published:** 1891-08-08

**Authors:** 


### Annotations.

**Beware of the Jam-Pot.**

Dr. H. M. Lawrence, of Hadlow, near Tunbridge, writes to a contemporary to warn the public against the use of glass jars for holding jam. Says Dr. Lawrence: "Twice within the last two months have my children found pieces of glass in their mouths, and fortunately got them out again; one piece was half an inch long and very sharp. If this has happened twice in so short a time to me, how often must it occur to others, and how often must children swallow a piece without knowing it? It makes me look back to the obscure cases of enteritis one has met with which could not be accounted for as to the cause. The jams my children had were from different makers, so the accident must be not uncommon." This suggestion of Dr. Lawrence's, that a certain number of obscure cases of enteritis may have had their origin in the swallowing, by children, of pieces of glass, or other sharp and irritating substances mixed with bought jams, deserves the most serious consideration. Every medical man of experience can remember cases of internal inflammatory affections in children, which have run a course more or less resembling that of typhoid fever, some of which have ended fatally. A certain proportion of those cases it has been impossible to account for. Dr. Lawrence's discovery opens a wide door for alarming conjecture. Children will swallow all kinds of things without detecting them, if there be no acute pain in the act of swallowing. Later on, when substances like pieces of broken glass have to make their way through the pylorus into the bowel, or along the narrow canal of the small intestine, their irritating presence may set up convulsive movements, with sharp pains and inflammatory action. Aperients, as is well known, may entirely fail to remove the offending substances; and their continued presence in the bowel rapidly increases the pain and inflammation, until death finally results. The danger associated with glass jars is very great. The jars themselves are so brittle that pieces are easily broken off; and the men, women, and children who work in jam factories mostly belong to such an ignorant and careless class that it is probable that they think little or nothing about breaking off little pieces from the jars in the hurry of their manipulation, and leaving them buried in the jam for the consumer to discover. Wise people will do well to have their jams made at home, and to store them in the old-fashioned earthenware jars. The risks which Dr. Lawrence has pointed out are very alarming to contemplate, at feast for medical parents who understand them. The manufacturers of jams for the market must alter their methods if their trade is not to be ruined.

**Nurse Training and Certification.**

The Lords' Committee have devoted themselves very earnestly and perseveringly to questions relating to nurses and nursing. There was one fact connected with training and certification which needed to be brought out very clearly; and the intelligent questionings of Lord Kimberley, Lord Cathcart, and Lord Thring have brought it out with the utmost definiteness and precision. The point is this: that a nurse's certificate, to be of any value to the public, must be a practical certificate; that is to say, a certificate which proves not only that a nurse has been trained at a hospital or hospitals for a certain length of time, but also that she is practically efficient. No one will deny that this fact of efficiency or non-efficiency is the most important of all the facts about the nurse's certificate. It is, in short, the facts of facts, the *sine qua non* of her worth as a nurse. But who is to give to each nurse this certificate of practical efficiency? Who, but her superiors who have trained and directed her in hospital? Who have been responsible for the work which she has done under their supervision? Who have experienced the worries arising from her failures, and the gratification and confidence resulting from her successes? Can anybody truly and rightly give a certificate of a particular nurse's practical efficiency, except those who have employed and seen her at work, and watched her in a responsible way for a sufficient length of time to know whether she is really a good nurse or not? It is plain then that whatever other certificates a nurse may possess or not possess, she must have one from the hospital where she was trained. If she cannot get this, all her other certificates are of little or no value, at any rate to the public. The noble lords, whose names we have mentioned, saw this point very clearly, and they made it clear to those who heard them, and to all who will read their report. Of course there is nothing in this which can be called an argument against general nurse registration. But there is everything in it to prove that nurse registration can only be properly carried out on certain definite lines, and that those lines must coincide with the concerted action of all the nurse-training schools. An outside body, acting independently of the nurse-training schools, and even in opposition to them, is like an invading army which digs a deep and impassable ditch between itself and its own base of operations; or it is like a tree, which, in order to make itself thrive the better, deliberately cuts off its own roots. There is a great schism in the nursing world, and until that schism is healed no progress can be made in the direction of general registration. The schismatics must repent them of their folly, and return to the fold of the nurse training schools. This is the absolutely indispensable condition of forward movement. If the schismatic association which has deserted the fold for so long refuses to take this quite indispensable step, it will simply persis in the ridiculous attempt to cut off its own nose to spite its face.

**Midwives' Registration.**

Some of the younger members of the medical profession are more impulsive than wise in dealing with questions which concern the public as much as, o more than, they do medical men. An earnest attempt has been made by Mr. Rathbone, M.P., supported and helped by some of the more statesmanlike leaders of the British Medical Association, to frame a measure for the registration of midwives, which should be at once a measure of safety for poor lying-in women and of justice to the members of the medical profession. That such a measure is imperatively called for in the interests of poor women no right-minded medical man can for a moment doubt. The Bill which was actually proposed failed to secure the support of a considerable portion of the profession. Some of those who opposed it, emboldened by their momentary success, had the temerity to attempt to carry a resolution at the Bournemouth meeting of the British Medical Association last week, which, if they had succeeded in carrying it, would have given a shock to all kindly-disposed men, both medical and lay. It was actually moved that the Parliamentary Bills Committee of the British Medical Association should be instructed to oppose any Bill whatever which might be introduced into Parliament having for its aim the registration of midwives by State authority. This motion even found a seconder. But Mr. Ernest Hart happily perceived that its effect, if carried, would be to drag the whole medical profession through the mud of public opprobrium. Mr. Hart pointed out how absurd and unnecessary such a resolution was, and showed that it would be a sufficient safeguarding of medical interests if the Parliamentary Bills Committee were instructed to give no furtherance to a Midwives' Registration Bill until it had received the approval of a majority of the branches of the British Medical Association. The resolution as originally proposed was rejected by a large majority, and Mr. Hart's amendment was carried with but one dissentient. It is eminently satisfactory to find that the British Medical Association as a body is much more rational and just than some of its would-be leaders.

